# Intelligence

**Published:** 1808-09

**Authors:** 


### INTELLIGENCE.

---

The COURT of the ROYAL COLLEGE OF SURGEONS have resolved, that the JACKSONIAN PRIZE SUBJECT, for the year 1809, for the Premium of 10l. to the Author of the best Dissertation on a Practical Subject in Surgery, be "Fractures of the Bones of the Trunk, and the Treatment of them."

Candidates must be Members of the College, not on the court of assistants.

The Dissertations must be in English; and the number and importance of facts in the composition will be considered their chief excellence.

Every Dissertation must have a Motto or Device of distinction; but no Dissertation, Motto, or Device, must be in the hand-writing of the Author.

Every Dissertation must be accompanied by a paper, sealed up, (not with the impression of the Author's seal) containing the name and residence of the Author, and having on the outside a Motto, (not in the hand-writing of the author), corresponding with the Motto or Device on the Dissertation.

The paper corresponding in Motto or Device with either of those affixed to the Dissertation, to the author of which the premium shall be adjudged, will necessarily be opened: the other papers will be returned unopened upon authenticated application, together with the Dissertations to which they shall respectively belong.

Dissertations must be addressed to the Secretary, and delivered at the College before Christmas-day 1809.

The adjudication will be in the month of April 1809.

Every unapproved Dissertation which shall remain three years unclaimed, will be burnt, together with the paper containing the name of the Author of such Dissertation, unopened, in the presence of the Jacksonian Committee.

It is judged proper to repeat, that the Prize Subject for the present year, 1808, is, "CANCER:" the Dissertations upon which must be delivered before Christmas-day, 1808.

---

Mr. WALKER, of Edinburgh, has ascertained, by numerous observations, that the *use of the medulla of bones*, which has so long puzzled physiologists, *is to regulate their internal growth.* A paper, detailing these observations, will soon appear in this Journal.

The same gentleman has observed, that the organ of voice in frogs consists of two membraneous bags, hitherto undescribed, placed one on each side of the tongue, immediately behind the curvature of the lower-jaw. These bags have each a circular aperture, which, by means of a sphincter, they can contract at pleasure; and it is by expressing air through them, that they produce their peculiar croaking noise.

Mr.

Mr. James Phœnix, of Liverpool, has found that there is a great difference between electrical shocks from a Leyden phial filled from the conductor in the common way, and those filled as follows. He stood on an insulated stool, laid one finger on the prime conductor, and filled the jar from the other; when, on receiving the shocks, he found them so considerably augmented, that two taken in this manner incommoded him more than a dozen in the common method.

Mr. George Singer is constructing an electrical apparatus with a cylinder eighteen inches in diameter, which, from experiments made with cylinders of nine and fifteen inches diameter, promises to afford at least equal intensity and regularity of action with with plate machines. A series of experiments will be shortly instituted on this apparatus, and their results communicated to the public.

A fish, called by the Spaniards the *curbinata*, the largest of which does not weigh more than two pounds, abounds in the river Oronoko, in South America. It is of an excellent flavour, but is less appreciated for its nutritive quality than for two stones lodged in the head, in the place which the brain ought to occupy. They have each the shape of an almond without the shell, and the brilliant colour of mother of pearl. These stones are bought for their weight in gold, on account of their specific virtue against a retention of urine. It is sufficient to take three grains finely powdered in a spoonful of wine or water, to cause an instant discharge; but too large a dose relaxes the muscles, and occasions an inability of retention.

The production of fire by the mere compression of atmospheric air, was a fact first observed about three years ago in France. This curious discovery has lately been applied to practical utility in this country, by means of an instrument which answers all the purposes of that well-known article in domestic economy, a tinder-box. It consists of a common syringe, about ten inches long, and not more than five eighths of inteanal bore. At the lower extremity it is furnished with a cap which serves as a chamber to receive the substance to be fired, and is attached to the instrument by a screw. Instead of this cap, a common stop-cock may be employed. To use the instrument, the cap is unscrewed, or the stop-cock turned, a small piece of amadou or common tinder, is placed in the chamber, and the cap is screwed on again. If the piston of the instrument be now depressed with as quick a motion as possible, the condensation of the air is so active, as to set the amadou on fire.

Mr. J. Wright has invented a portable artificial horizon for taking altitudes either by sea or land. With this instrument and a quadrant, varying from those of the usual construction in having a larger horizon glass, the silver surface larger, and a different sight vane, the meridian altitudes of all bright stars, as they come to the the meridian may be taken; by which means the latitude might frequently be found by observations at night, and with as much ease as by the sun at noon day; the altitudes of the moon and stars to correct the lunar problem for the longitude will also be more accurately and easily taken with it. For altitudes of the sun or moon, and for all terrestrial objects, an octant of the ordinary construction will answer every purpose.

The Medical Lectures in the UNIVERSITY OF GLASGOW, will begin on Tuesday November 1, at the following hours.—Dietetics, Materia Medica, and Pharmacy, by Dr. MILLAR, at ten o'clock in the forenoon.—Midwifery, by Mr. TOWERS, at eleven.—Theory and Practice of Physic, by Dr. FREER, at twelve.—Anatomy and Surgery, by Dr. JEFFRAY, at two o'clock in the afternoon.—Chemistry and Chemical Pharmacy, by Dr. CLEGHORN, at seven.—Clinical Lectures on the cases of patients in the Royal Infirmary, on Thursday evening the 10th of November, at six.—Dr. BROWN will commence his Lectures on Botany about the beginning of May next.

MEDICAL THEATRE, LONDON HOSPITAL.—The following Lectures will commence on October 1st. viz.—Theory and Practice of Physic, by Dr. COOKE, and Dr. YELLOLY.—Chemistry, by Dr. YELLOLY.—Theory and Practice of Midwifery, by Dr. DENNISON and Dr. Byam DENNISON.—Clinical Lectures on Surgical Cases, by Sir W. BLIZARD, and Mr. Thos. BLIZARD. Further particulars may be known by applying to Mr. PRICE, Apothecary at the Hospital.

ST. BARTHOLOMEW'S HOSPITAL.—The Autumnal Courses of Lectures will commence on Saturday, October 1, at two o'clock, in the Medical Theatre of this Hospital, in the following manner: On the Theory and Practice of Medicine, by Dr. ROBERTS and Dr. POWELL.—On Anatomy and Physiology, by Mr. ABERNETHY.—Anatomical Demonstrations, by Mr. LAWRENCE.—On Comparative Anatomy, by Mr. MACARTNEY.—On the Theory and Practice of Surgery, by Mr. ABERNETHY.—On the Theory and Practice of Midwifery, by Dr. THYNNE.—On Chemistry, by Dr. HUE.

The Autumnal Course of Lectures at ST. THOMAS'S and GUY'S HOSPITALS, will commence the beginning of October.

At ST. THOMAS'S.—Anatomy, and the Operations of Surgery, by Mr. CLINE and Mr. COOPER.—Principles and Practice of Surgery, by Mr. COOPER.

At GUY'S.—Practice of Medicine, by Dr. BABINGTON and Dr. CURRY.—Chemistry, by Dr. BABINGTON, Dr. MARCET, and Mr. ALLEN.—Experimental Philosophy, by Mr. ALLEN.—Theory of Medicine, and Materia Medica, by Drs. CURRY and CHOLMELEY.—Midwifery, and Diseases of Women and Children, by Dr. HAIGHTON.—Physiology, or Laws of the Animal Œconomy, by Dr. HAIGHTON.—Clinical Lectures on Select Medical Cases,

Cases, by Dr. BABINGTON, Dr. CURRY, and Dr. MARCET.—Structure and Diseases of the Teeth, by Mr. FOX.—These several Lectures are so arranged, that no two of them interfere in the hours of attendance; and the whole, together with the Lectures on Anatomy, and those on the Principles and Practice of Surgery, given at the Theatre of St. Thomas's Hospital adjoining, is calculated to form a complete Course of Medical and Chirurgical Instructions.

Mr. BROOKES's Spring Course of Lectures on Anatomy, Physiology, and Surgery, will commence on Saturday, October the 1st, at two o'clock in the Afternoon, at the Theatre of Anatomy, Blenheim-Street, Great Marlborough-Street.

Mr. TAUNTON's Autumnal Course of Lectures on Anatomy, Physiology, Pathology, and Surgery, will commence on Saturday, Oct. 1, 1808. Further Particulars may be learned on application to Mr. Taunton, Greville Street, Hatton Garden.

Mr. THOMAS's Lectures on the Principles and Operations of Surgery, will commence early in October, as usual. A Prospectus may be had at his house, Leicester-place, or at the Theatre of Anatomy, in Windmill-street.

Mr. WILSON's Lectures on Anatomy, Physiology, Pathology, and Surgery, will begin on Saturday, Oct. 1, at two o'clock in the afternoon as usual. A plan of the Course, with the terms of Attendance, may be had by applying at Mr. Wilson's house, in Great Windmill Street.

Mr. ACCUM's Lectures on Experimental Chemistry and Analytical Mineralogy, commence at the Chemical Laboratory, Compton-street, Soho, October the 18th. The Lectures on Experimental Chemistry, comprise the Practical Operations of the Scientific Laboratory; general Rules to be observed in the performance of Experiments, and summary Experimental Elucidations of the Science of Chemical Philosophy. The Lectures on Analytical Mineralogy devolve to the Art of distinguishing Minerals, the Modes of examining them by Chemical Agencies; and general process of Analysis, with a summary View of Mineralogical Science, and its application to the useful Arts and Manufactures.

Dr. BUXTON will, on Monday Oct. 3, commence a Course of Lectures on the Theory and Practice of Medicine, and one on Materia Medica, at the London Hospital.

Mr. CARPUE will commence his Lectures on Anatomy, Surgery, &c. on the 1st. of October.—Particulars may be known by applying to Mr. Carpue, No. 50, Dean Street, Soho.

Dr. CLARKE and Mr. CLARKE, will begin their Winter Course of Lectures on Midwifery and the Diseases of Women and Children, on Wednesday Oct. 5. The Lectures are read every day at the house of Mr. Clarke, No. 10, Upper John Street, Golden Square, from a quarter past ten o'clock in the morning, till a quarter quarter past eleven, for the convenience of Students attending the Hospitals. For particulars, apply to Dr. Clarke, No. 1, New Burlington Street; or to Mr. Clarke, No. 10, Upper John Street, Golden Square.

Dr. Clough, No. 68, Berner's Street, will, on Monday the 17th of October, at ten o'clock in the morning, commence his Course of Lectures on Puerperal Anatomy, Physiology, and Pathology. A Syllabus and Prospectus of which, may be had of Dr. Clough, as above; J. Callow, Crown Court, Soho; and at the Mary-le-bone Dispensary, Welbeck Street, Cavendish Square. An Evening Course, for the convenience of such Students as may be otherwise engaged in Anatomical pursuits, will be given on Tuesdays, Thursdays, and Saturdays, at seven. The first Lecture commencing on the Evening of the 18th Instant.

Dr. Clutterbuck, Member of the Royal College of Physicians, and one of the Physicians to the General Dispensary, will begin his Winter Course of Lectures on the Theory and Practice of Physic, the Principles of Pharmacy, and the Materia Medica, on Tuesday October 4, at ten o'clock in the Morning: to be continued daily (Saturdays excepted) at the same hour, at the General Dispensary, Aldersgate Street, near St. Bartholomew's Hospital.——Clinical Lectures, by Dr. Clutterbuck and Dr. Birkbeck, on the most interresting cases occurring in the Practice of the Dispensary, will be given *gratis* to the pupils of the Class, on Saturdays, through the season.—Further particulars, with a Prospectus, may be had on application at the Dispensary, or at No. 1, Crescent, New Bridge Street.

On Saturday, Oct. 1st, Dr. Dennison and Dr. Byam Dennison, will commence their Lectures on the Theory and Practice of Midwifery and the Diseases of Women and Children, at the London Hospital. For Particulars, apply at the Doctor's house, No. 37, Broad Street Buildings; or to Mr. Price, Apothecary, at the Hospital.

Mr. Moor, Surgeon Dentist to Her Royal Highness the Duchess of York, will commence a course of Lectures on the Structure and Diseases of the Teeth, on the 27th of October, in which will be explained the complete Practice of the Dentist.—Further Particulars may be known at his House, No. 6, Palsgrave Place, Temple.

Dr. J. F. Davis, of Bath, has in the press an Inquiry into the Symptoms of Carditis, or the Inflammation of the Heart, illustrated by Cases and Dissections. It is Dr. Davis's design to show, that the Faculty have been mistaken with regard to the symptoms of this disease; that it is probably of more frequent occurrence than has been supposed; and that, contrary to the assertions of our best systematic writers, there are circumstances by which it may sometimes be distinguished in practice.

